# Cost‐benefit analysis of acoustic recorders as a solution to sampling challenges experienced monitoring cryptic species

**DOI:** 10.1002/ece3.4199

**Published:** 2018-06-14

**Authors:** Emma M. Williams, Colin F. J. O'Donnell, Doug P. Armstrong

**Affiliations:** ^1^ Matuku Ecology Christchurch New Zealand; ^2^ Wildlife Ecology Group Massey University Palmerston North New Zealand; ^3^ Department of Conservation Biodiversity Group Christchurch New Zealand

**Keywords:** acoustic monitoring, Australasian bittern, *Botaurus poiciloptilus*, cryptic, detectability, monitoring, New Zealand, surveys

## Abstract

The inferences that can be made from any study are limited by the quality of the sampling design. By bad luck, when monitoring species that are difficult to detect (cryptic), sampling designs become dictated by what is feasible rather than what is desired. We calibrated and conducted a cost‐benefit analysis of four acoustic recorder options that were being considered as potential solutions to several sampling restrictions experienced while monitoring the Australasian bittern, a cryptic wetland bird. Such sampling restrictions are commonly experienced while monitoring many different endangered species, particularly those that are cryptic. The recorder options included mono and stereo devices, with two sound file processing options (visual and audible analysis). Recording devices provided call‐count data similar to those collected by field observers but at a fraction of the cost, which meant that “idealistic” sampling regimes, previously thought to be too expensive, became feasible for bitterns. Our study is one of the few to assess the monetary value of recording devices in the context of data quality, allowing trade‐offs (and potential solutions) commonly experienced while monitoring cryptic endangered species to be shown and compared more clearly. The ability to overcome challenges of monitoring cryptic species in this way increases research possibilities for data deficient species and is applicable to any species with similar monitoring challenges.

## 1 INTRODUCTION

Sampling designs dictate the strength of the inferences that can be made about a population of interest (Lambert et al., 2009; Thompson, White, & Gowan, 1998; Williams, Nichols, & Conroy, 2002). Thus, great care must be taken when designing monitoring programs to ensure that sampling regimes are appropriate and representative of the population and species of interest. Producing good sampling designs for cryptic species that are difficult to detect is particularly problematic, as detections are sparse and sampling areas are often large, resulting in monitoring programs that quickly become costly in terms of time and money (Thompson, 2004). In general, when the cost of sampling an entire area of interest is too great, researchers subsample by defining sampling units considered representative of the area that has not been visited (Caughley, 1977; Thompson et al., 1998; Williams et al., 2002). In this way, biases caused by spatial variation can be limited using random‐ or stratified‐sampling procedures. By bad luck, subsampling is also problematic with cryptic species, as work on these species is also logistically restricted. Such restrictions can prevent the application of many statistically valid sampling procedures, resulting in biased inferences or small sample sizes (Williams, 2016).

Automated acoustic recording devices are being used increasingly to overcome some of the logistical challenges experienced while sampling cryptic species for both survey and monitoring programs (e.g., Frommolt, 2017; Frommolt & Tauchert, 2014). For example, calls of Cory's shearwaters (*Calonectris diomedea*) are most detectable at night. This, combined with their tendencies to breed on inaccessible cliff faces on remote islands, complicates monitoring (Goh, 2001). Acoustic recorders can be used to overcome these restrictions (Goh, 2001). In the same way, recorders have been used to overcome diel and seasonal restrictions in detecting black drum (*Pogonias cromis*) and spatial restrictions associated with monitoring African forest elephants (*Loxodonta africana cyclotis*) in large remote areas (Locascio & Mann, 2011; Thompson, Schwager, Payne, & Turkalo, 2009). However, the costs and benefits of using acoustic recorders compared to traditional sampling techniques require investigation.

In New Zealand, the Department of Conservation has developed two affordable recording devices for monitoring wildlife: one stereo option that records calls in MP3 format and a mono option that records files in WAV format. In theory, the advantage of stereo recordings is that an observer listening to files should be able to determine the number of individuals calling, whereas only a simple index of calls per unit time can be recorded on mono recorders (Acevedo & Villanueva‐Rivera, 2006). In addition, there are currently several options for processing recordings. Sound files can be: (a) manually processed by listening or visually examining files for evidence of calls on spectrograms; or (b) automatically processed using software trained to identify unique sound shapes associated with calls of the target species (Brandes, 2008; Joshi, Mulder, & Rowe, 2017). Here, we tested four possible manual options for monitoring wildlife calls using recording devices: (a) stereo recordings processed visually (STEREO‐VISUAL), (b) mono recordings processed visually (MONO‐VISUAL), (c) stereo recordings processed audibly (STEREO‐AUDIBLE), and (d) mono recordings processed audibly (MONO‐AUDIBLE).

For recorders to be the preferred option (over field observers) for monitoring cryptic species, then: (a) The number of calls detected by recorders would need to be comparable or better than the number detected by field observers (to facilitate replacement of observers); and (b) recorders would have to be cost‐effective and practical in terms of increasing coverage, sampling multiple locations concurrently, and sampling at specified “optimum” times with greater repeatability. In addition, in some circumstances it may be advantageous to obtain estimates of the number of calling individuals. For example, number of individuals may provide a better measure of population change compared with the index measure of the number of call sequences. To a certain degree, field observers can distinguish between calling individuals during counts, so theoretically this should be possible using stereo devices. As such, there is a need to investigate whether stereo recordings processed audibly can be used to measure the number of calling individuals.

This study aims to answer three questions: (a) How well do a number of calls detected with each recording option correlate with the number of calls detected by observers? (b) Is there a predictable relationship between the number of individuals detected listening to stereo recordings compared with the number detected by field observers? and (c) Are recording devices more cost‐effective in terms of money and effort for monitoring?

## 2 MATERIALS AND METHODS

### 2.1 Study species

**Figure 1:**
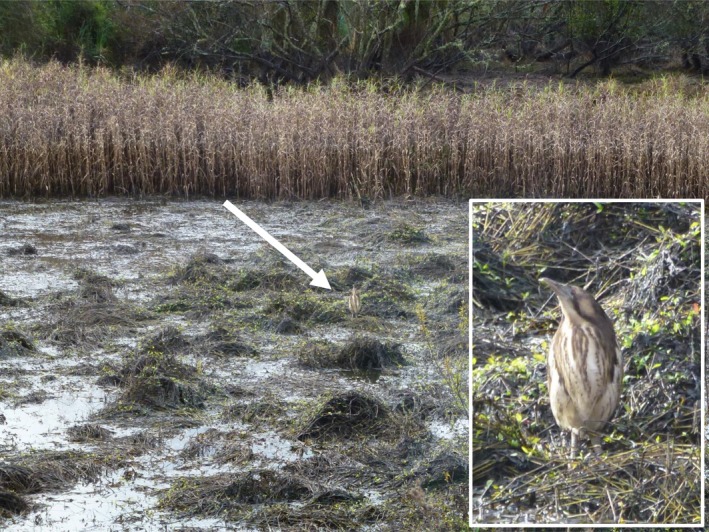
The Australasian bittern (inset), Whangamarino wetland, 2016. The cryptic plumage and behaviors of this species means individuals are difficult to see in their environment even when standing exposed in the open

A typical example of these challenges can be found with the Australasian bittern (*Botaurus poiciloptilus*; matuku) an endangered cryptic wetland bird (BirdLife International 2018). Bitterns present an appropriate case study for monitoring a cryptic species because their plumage makes them difficult to see in their environment (Figure 1) and large areas of their habitat are inaccessible, restricting standard sampling practices at many sites. Yet, conservation managers are interested in identifying the causes of decline of bitterns so that key habitats can be managed to reverse population declines (Kushlan, 2007; O'Donnell, 2011). As such, reliable monitoring methods are required to evaluate the effectiveness of management practices for this species. Acoustic surveys have potential in this regard.

Male Australasian bitterns produce a series of unique calls or “booms,” hereafter known as a call sequence, that are often, but not always, preceded by a series of inhalations. Each boom within the call sequence is made up of a first element and main element as described by Gilbert, McGregor, and Tyler (1994). Call‐counts of male bitterns are currently thought to be the most feasible means of monitoring populations (O'Donnell & Williams, 2015; White, Purps, & Alsbury, 2006; Williams, 2016). However, bittern calling‐rates fluctuate in response to temporal and environmental factors, creating high variability in detection rates (Williams, 2016). Standardizing monitoring protocols reduces this variability but introduces logistic restrictions, creating the trade‐off described above, where it becomes difficult to adequately cover an extensive sample area while retaining valid sampling procedures. Current trade‐offs experienced while monitoring bitterns include:

1. Detectability is optimum during a short sampling window at the start of the breeding season (Williams, 2016). The shorter the window the more restrictive the sampling becomes, reducing the area that can be covered.
2. Detectability is highest one hour before sunrise or the first 30 min after sunset (Williams, 2016), a time when few people are present in wetland environments. To adequately monitor bitterns, field staff need to work split shifts, mostly in darkness hours. This is taxing on staff, and has greater health and safety implications compared to normal work regimes (Witmer, 2005). Thus, it may be difficult to persuade staff and/or volunteer groups to conduct counts at these times (Gibbs & Melvin, 1993). These restrictions limit our ability to sample multiple areas concurrently. Such challenges are also commonly experienced with nocturnal species.
3. Sampling is limited to accessible areas. Large wetlands are time‐consuming and costly to access. Sampling for bitterns using statistically sound regimes is either not possible or produces small sample sizes. Sampling using regimes that are more affordable, for example, sampling accessible areas, can be biased or have insufficient power (Gibbs & Melvin, 1997; Robbins, 1986). These problems are amplified now that the optimum time to detect bitterns has been identified as one hour before sunrise (Williams, 2016).

### 2.2 Data collection

**Figure 2:**
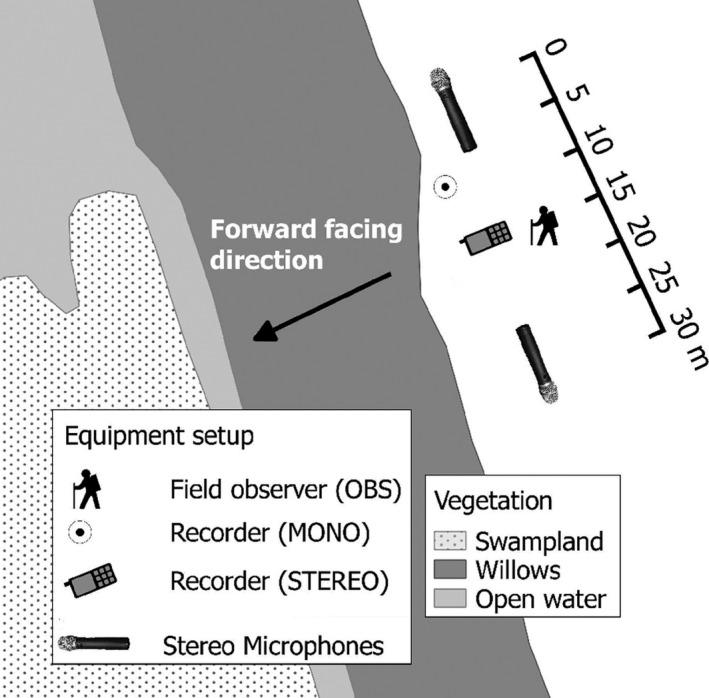
Typical placement of the two recording devices (STEREO and MONO) trialed in relation to the field observer (OBS) at Whangamarino wetland in 2010

A total of 137 call‐counts were conducted at Whangamarino wetland, New Zealand (>7,100 ha), by field observers from September to November 2010. As well as having an observer in the field (OBS) during call‐counts, each count was recorded using at least one of two different types of recording units (MONO and STEREO). The availability of recorders depended upon other projects, and therefore differed throughout the season. Wherever possible, all three means of data collection (OBS, MONO and STEREO) were run concurrently to allow direct comparisons (Figure 2). However, of the 137 call‐counts conducted, direct comparisons were only possible for 43 counts (OBS, MONO and STEREO), a further 80 involved an observer and mono recorder (OBS and MONO), and the last 14 involved an observer and stereo recorder (OBS and STEREO).

Each call‐count lasted 15 min, after which the observer moved on to a consecutive station. Stations were positioned ≥400 m apart in accessible areas of the wetland. During counts, field observers noted all bittern call sequences and made an assessment of how many bitterns were heard calling within a count using a combination of call direction and call characteristics (call volume and number of booms in sequence). Each time a bittern was heard, observers made a judgment as to whether the call was from a new bird, or one that had been previously heard within the count. A call sequence was considered to be a new bird if any of the following was true (Pierce, 2004): The bearing of the new call was

1. >10° different from the bearing of any call previously heard;
2. within 10° of another call but fell into a different volume category;
3. within 10° of another call, but consistently had a different number of booms within its sequence compared to the previous call.

Where uncertainty existed as to whether one or two birds were calling, only one bird was recorded. As a result, the number of individual birds detected by observers was considered to represent a minimum number of individuals detected.

Mono recorders (Department of Conservation Ver. B.2), were fixed at a height of 1.5 m (approximately ear height, to provide the best comparability with the field observer) and as close to the observer as possible (within 15 m, Figure 2). Timers on mono units were preset to record automatically for two daily observation periods designed to contain peak booming activity, each spanning five hours (03:00–08:00 including sunrise and 17:30–22:30 incorporating sunset). Observers verbally marked the beginning and end of their counts on the recorders so that exact times on sound files could be matched correctly to field counts. Sound files were then cut to exact count times later using the software Audacity (Version 2.0.5).

Stereo recording units, consisting of an Olympus LS‐10 recording device with two external microphones and an external 4× D‐cell power source, were positioned 15 m away from the observer at a height of 1.5 m and a bearing of 90° from the observer's forward‐facing direction (Figure 2). Each stereo microphone was manually switched on/off by the observer at the beginning/end of each count. Units were programmed to record at 128 kbps (maximum recorder level), with a microphone sensitivity of high and no limiter.

### 2.3 Data analysis

#### 2.3.1 Sound file analysis

Each sound file was analyzed twice, once visually, and then again audibly by the same processor. To prevent any bias caused by a processor's prior knowledge of a sound file's recording time, location and number of birds present, sound files were duplicated before processing and given a unique random identifier. These were matched to original attributes of the sound file only after processing had finished. To analyze files visually, the spectrograms of each file were examined looking for evidence of booms in Raven Pro 1.4 using the following view settings: *Y*‐axis <900 Hz, *X*‐axis = 0–30 s and sharpness = 2,792. Prior to visual analysis, stereo sound files were converted from their original MP3 format to WAV format so that they could be opened in Raven Pro 1.4. This was done using the website ‘online‐convert.com” (QaamGo Media GmbH 2012) with “no change” specified for the bit resolution, sampling rate and audio channel. For audible analysis, each file was listened to in real time in VLC media player with volume settings of 130% for mono recordings and 100% for stereo recordings. Audible analysis was performed using original file formats (stereo = MP3, mono = WAV). Listening volumes differed between mono and stereo devices because there was a noticeable difference between the loudness of mono and stereo recordings when they were listened to at the same volume setting. Sound file analysis was conducted on an ASUS A8H notebook laptop using the same set of Sennheiser HD201 headphones.

The number of call sequences detected from each recorder option was then compared with the number of call sequences detected by the field observers within the same count. This was done using a Spearman's correlation test, with the function “cor.test” in Program R (R Development Core Team 2010) because data were non‐normal (approximately Poisson distributed) (Hauke & Kossowski, 2011).

Four different observers were used to collect OBS data, while only one person was used to process sound files. Despite this, it was not deemed necessary to investigate whether an observer effect occurred with OBS data because data from a closely related study, that used the same observers as this study, demonstrated there was no observer effect (Williams, 2016).

In addition, the number of individual birds heard on stereo sound files was estimated during audible processing. To do this, an assessment was made of volume (low, med, high) and direction (left ear/right ear) for each call sequence. A call sequence was then classed as being from a new individual if the combination of volume and direction differed from calls previously recorded. Where uncertainty existed as to whether one or two birds were calling, only one bird was recorded. As a result, the number of individual birds detected audibly on stereo recordings can also be considered an estimate of minimum number of individuals detected. The strength of the association between number of individual birds calling on stereo sound files and number of birds heard by observers in the field was assessed using Spearman's correlation (rho).

#### 2.3.2 Cost‐benefit analysis

To determine whether recorders were more cost‐effective than observers, monetary costs were quantified for each option (OBS, MONO‐AUDIBLE, MONO‐VISUAL, STEREO‐AUDIBLE, STEREO‐VISUAL). This analysis was based on a sampling regime shown to have sufficient power (>80%) to detect a change in bittern calling‐rates of ±10% at Whangamarino wetland (Williams, 2016). This regime was designed to measure success (prevention of a decline in the bittern population) of a new management intervention (predator control) at Whangamarino wetland using conducting call‐counts lasting 15 min, at 40 stations (20 trapped, 20 untrapped), on six consecutive nights during the bittern breeding season. Costs included the initial purchase price of equipment, vehicle costs and staff wages. Wages ($NZ 25/hr) were allocated for the time traveling to and from the site, conducting call‐counts, analyzing sound files and setting up equipment. The purchase price of equipment and estimates of time/effort required were based on those incurred while monitoring bitterns at Whangamarino wetland in previous years, as well as the field costs incurred during this study (using the methods outlined above).

For MONO options, it was assumed that the initial purchase price of each device was $NZ 300, one device was needed per station (allowing simultaneous sampling), and that each device took 2.5‐min to program prior to deployment and 2‐min to secure in place at each station. Transport time around the wetland (accessing all stations) was assumed to be 45 min (0.75 hr) and stations were spread evenly across the sample area. Two trips around the wetland were required, one to deploy units, and the other to retrieve them. For transport to and from the site, a driving time of 2 hr return (130 km, Department of Conservation Te Rapa office to Whangamarino wetland and back) was assumed per trip. Vehicle costs were assumed to be $NZ 0.77 per km (Standard mileage rate, Inland Revenue Department 2014).

For STEREO options, the initial purchase price of $600 was assumed per device, and one device was needed per station. Each STEREO device took approximately 3 min to program prior to deployment and 10 min to deploy. STEREO devices took longer to deploy than MONO because there were three parts to assemble and secure (two microphones and a recording unit) rather than one. In addition, microphones were placed 15 m apart, so some of this extra time involved checking distances with the tape measure. Deployment costs were accrued per sampling occasion because, unlike MONO devices, STEREO devices did not have timers, and batteries lasted <24 hr. This meant that devices needed to be serviced before each sampling occasion. In the same way, it was assumed that transport time around the wetland, driving times to the site and vehicle costs were the same for STEREO as MONO but separate trips were required to service STEREO devices before each sampling period. Costs of sound file analysis were the same across recording devices but differed between VISUAL and AUDIBLE options. As a result, times of 0.1 and 0.25 hr per sound file (i.e., 6 min/file and 15 min/file) were assumed for analyzing VISUAL and AUDIBLE options respectively.

For OBS options, it was assumed that all observers would attend a 0.5 hr briefing prior to each sampling occasion (OBS equivalent of “programming time”). Separate briefings were assumed per sampling occasion because in the past the same observers were rarely available for all occasions. One observer was assumed per station (to allow simultaneous sampling). Deployment time was assumed to be minimal for observers because in prior years they were dropped off by vehicle directly at count stations. This meant all they needed to do before starting was orientate themselves and organize their field sheets (<1 min). However, unlike devices, staff preferred to be paid for their time in the wetland, so the cost of conducting counts was assumed to be equal to the count duration multiplied by the number of counts and the number of sampling occasions. The time it took to move around the wetland, drive to and from the site, and vehicle costs, were assumed to be the same as for recorder options except that a complete trip was required for each sampling period. In addition, unlike recorder options, overall traveling costs were assumed to accrue per observer because in prior years staff were paid travel time. An extra vehicle (incurring separate vehicle costs) was assumed for every five observers used.

To put these costs into perspective, each option was also assessed with respect to what could be achieved (values). Three commonly used values were considered; these included: (a) ability to measure change in call‐rate across time (as a surrogate for an index of abundance), (b) ability to measure change in number of calling males as an index of abundance, (c) ability to provide opportunities to engage local volunteers and landowners in conservation activities.

## 3 RESULTS

### 3.1 Comparisons among devices

**Figure 3:**
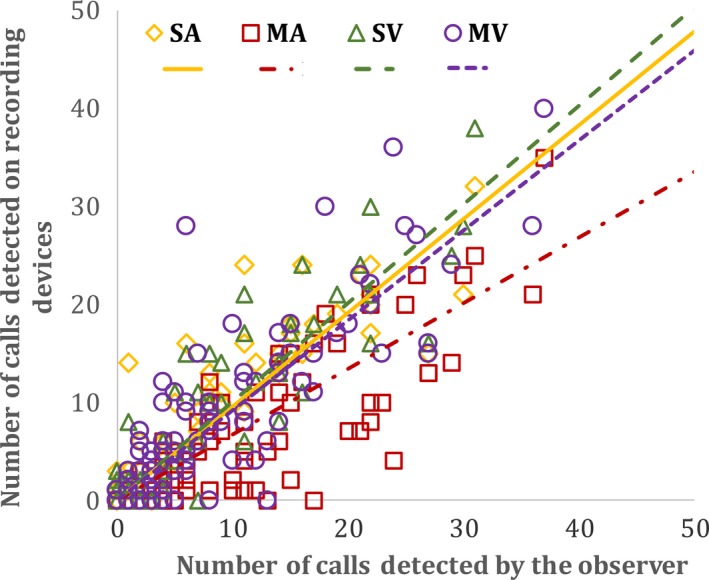
Relationship between number of Australasian bittern calls detected at Whangamarino wetland between September and November 2010 using four recording device options and numbers detected by the field observer, where SV = sound files analyzed visually and produced with stereo recorders (STEREO‐VISUAL), MV = sound files analyzed visually and produced with mono recorders (MONO‐VISUAL), SA = audibly analyzed sound files produced using a stereo recorder (STEREO‐AUDIBLE), MA = audibly analyzed sound files produced by a mono recorder (MONO‐AUDIBLE). All intercepts have been forced through the origin

**Figure 4:**
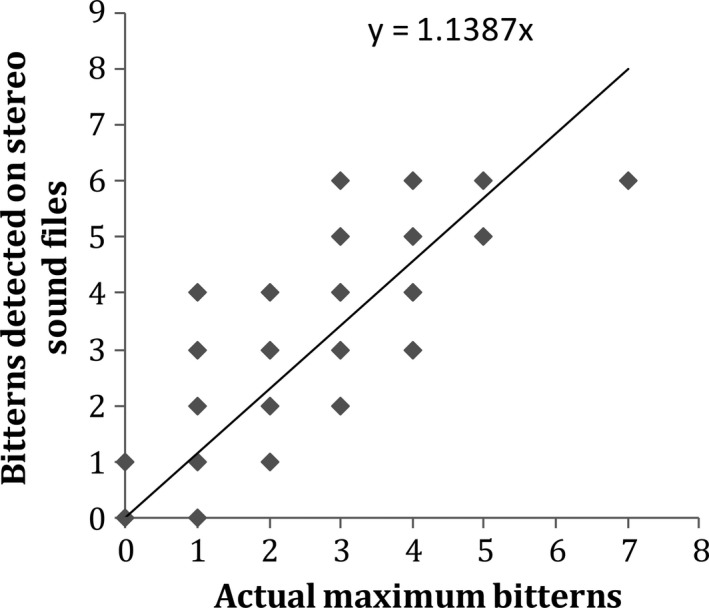
A comparison between the number of individual Australasian bitterns detected audibly on sound files and the number detected by field observers at Whangamarino wetland between September and November 2010. Actual maximum bitterns are the maximum number of bitterns detected by field observers within a 15‐min call‐count at each station. Here, the intercept has been forced through the origin

The number of bittern calls detected using all four recording device options were highly correlated with the number of calls detected by the field observers (rho > 0.80). This suggested strong positive associations between the number of calls detected on devices and those detected by observers, regardless of option used (Table 1, Figure 3). However, despite similar correlations across recorder options, associations between devices and observers were slightly stronger for stereo options compared with mono options (0.89 > 0.84, Table 1), and slopes of visual options were closer to a 1:1 ratio compared with audible options (STEREO‐VISUAL = 1.01 ± *SE* 0.04 > STEREO‐AUDIBLE = 0.96 ± *SE* 0.04; MONO‐VISUAL = 0.92 ± *SE* 0.03 > MONO‐AUDIBLE = 0.67 ± *SE* 0.03, Table 1, Figure 3). In the same way, the association between the number of calling bitterns detected through audible analysis of stereo files was also promising, showing a strong correlation with the number of bitterns detected by observers in the field (rho = 0.76, slope = 1.14, *p* < 0.01, Figure 4).

**Table 1:** Spearman's correlation coefficients (rho) showing associations between number of Australasian bittern calls detected using four recording options and the number of calls detected by field observers positioned in close proximity to each device. *Z* values were obtained by applying Fisher's transformation to the correlation coefficient (rho)

Recorder	Analysis	*Z*	rho	*p*	*df*	*N*	Slope	CI 95%Lower	CI 95%Upper
Mono	Audible	1.22	0.84	0.00	122	124	0.67	0.62	0.72
Mono	Visual	1.26	0.85	0.00	115	117	0.92	0.85	0.98
Stereo	Audible	1.47	0.90	0.00	46	48	0.96	0.87	1.044
Stereo	Visual	1.47	0.90	0.00	54	56	1.01	0.93	1.08

### 3.2 Cost comparisons

Cost analysis for all options showed that MONO‐VISUAL ($13,925) were the least expensive option followed by STEREO‐VISUAL ($28,113) provided 2 years of costs were considered (Table 2). Both recorder device options were cheaper than the observer option (OBS, $51,643). Regardless of device used, VISUAL analysis options (STEREO = $28,113; MONO = $13,925) were cheaper than AUDIBLE analysis options (STEREO = $29,913; MONO = $15,725). Most of the costs associated with recording devices involved the purchase of the equipment (STEREO > 88% of first‐year costs, MONO > 89% of first‐year costs), which meant that these options were particularly cheap in subsequent years (STEREO < $2,933, MONO < $1,863). Much of the cost with the observer option (OBS) involved paying people to sit in a vehicle and vehicle related costs ($12,000 + $4,500 + $4,805 = $21,305 per year) (Table 2).

**Table 2:** Costs of five options considered for monitoring Australasian bitterns using a sampling regime designed to determine the success (prevention of a decline in bittern numbers) of a management intervention (predator control) at Whangamarino wetland. Options include combinations involving field observers (OBS), use of recording devices (one STEREO and MONO options), and two sound file analysis techniques (VISUAL and AUDIBLE)

	Costs	MONO		STEREO		OBS
		VISUAL	AUDIBLE	VISUAL	AUDIBLE	
Year One	Purchase	12,000	12,000	24,000	24,000	0
	Processing/counting	600	1,500	600	1,500	1,500
	Deployment	33	33	167	167	17
	Driving to/from site	50	50	300	300	12,000
	Driving around site	38	38	113	113	4,500
	Programming	42	42	300	300	3,000
	Vehicle running	200	200	601	601	4,805
Total ($)		12,963	13,863	26,080	26,980	25,821
Year Two	Purchase	0	0	0	0	0
	Processing/counting	600	1,500	600	1,500	1,500
	Deployment	33	33	167	167	17
	Driving to/from site	50	50	300	300	12,000
	Driving around site	38	38	113	113	4,500
	Programming	42	42	300	300	3,000
	Vehicle running	200	200	554	554	4,805
Total (NZ$)		963	1,863	2,033	2,933	25,821
Cumulative costs (NZ$ over 2 years)		13,925	15,725	28,113	29,913	51,643

When monitoring values were also considered as part of the cost analysis, mono options were the least diverse (could only measure call‐rate) but were also the cheapest (Table 3). Stereo options were cost‐effective if the number of individual bitterns was an important measure, but public participation was not valued (Table 3). Options using field observers were the most diverse as observers could be used to achieve all three values. However, depending upon the circumstances, the threefold increase in costs associated with using observers rather than mono options may not justify this diversity (Table 3).

**Table 3:** Performance of options in relation to three values that managers may wish to achieve while monitoring Australasian bitterns: (a) Measure change in call‐rate across time (as a surrogate for an index of abundance), (b) Measure of change in number of calling males as an index of abundance, (c) Engage local volunteers and landowners. Values that are easily achievable are denoted with a double tick (✓✓), values that could be achieved with some work are shown with single ticks (✓), and those currently unachievable are shown with a cross (✗). Options considered include involving field observers (OBS), the use of recording devices (STEREO or MONO), and two sound file analysis techniques (VISUAL and AUDIBLE)

Values	MONO		STEREO		OBS
	VISUAL	AUDIBLE	VISUAL	AUDIBLE	
Index using call‐rate	✓✓	✓✓	✓✓	✓✓	✓✓
Estimate of numbers	✗	✗	✗	✓	✓✓
Public participation	✗	✗	✗	✗	✓✓
Costs (NZ$ over 2 years)	13,925	15,725	28,113	29,913	51,643

## 4 DISCUSSION

Several studies have shown that the quantity and quality of monitoring data can be improved by replacing field observers with recording devices (Acevedo & Villanueva‐Rivera, 2006). A few authors have demonstrated the value of recording devices for solving problems in detecting cryptic species, including wetland birds, and bitterns in particular (Frommolt & Tauchert, 2014). For example, Zwart, Baker, McGowan, and Whittingham (2014) showed that recorders could be used to increase detection probabilities for cryptic European nightjars (*Caprimulgus europaeus*). The probability of detecting nightjars improved with recording devices because, unlike field observers, recorders can be left in the field for extended periods, allowing longer count durations for similar costs. This extension in count duration means that nightjars that vocalise in short bursts are still detected (<10 min/hr, Zwart et al., 2014). Similarly recording devices have been used with cryptic species to improve detection probability estimation for occupancy studies (Gorresen, Miles, Todd, Bonaccorso, & Weller, 2008). However, this is the first study to show in monetary terms that devices can be used to achieve a desired sampling regime for monitoring a cryptic species that would otherwise not be achievable, therefore potentially allowing a species to be monitored with sufficient power.

The comparison of the two recording devices, and two sound file processing options against field observers, made in this study, showed that any recording device option could be used instead of field observers if desired. In all four cases, data collected using recording devices was comparable with that collected by field observers (number of calls and number of individuals). Yet, costs associated with recording devices were far lower than those of the field observer (Recording devices < $NZ 30,000, Observers > $NZ 51,000). As a result, the decision of which option to use can be based upon which values are the most important given a project's monitoring objectives.

In circumstances where costs and site accessibility are not key factors limiting monitoring then the use of observers would be the preferred option. This is because observer options are an effective way to get local communities involved and provide measures of both call‐rate and the number of individuals calling (Table 3). In addition results are instantaneous as no sound files need to be processed. However, funds are rarely superfluous when it comes to conservation, so if all three values are not required then the 73% increase in costs using observers may not be justified (MONO‐VISUAL = $13,925; OBS = $51,643). Most of the costs associated with observer options were incurred as wages and because of needing to make multiple trips to the wetland. These costs could be reduced by using local volunteer groups. However, in the case of the sampling regime tested here, temporal and spatial variation in calling‐rates meant 40 stations were required to achieve sufficient power to detect a change of 10% in calling‐rate (Williams, 2016) and it is likely that managers would find it difficult to organize enough local volunteers (pers. comm., Matthew Brady, Department of Conservation ranger). In addition, the time and effort required to get volunteers safely into the heart of an inaccessible wetland for a short 15 min count would be inefficient and nonsensical. As such, observer options are best suited to areas where wetlands occur in small accessible pockets and local project buy‐in is necessary or high.

If the number of individual bitterns is the desired monitoring measure but public participation is not necessary (or possible) then it would be better to take the 46% saving in costs and use the stereo‐audible option (STEREO‐AUDIBLE = $28,113; OBS = $51,643). Like observer options, deployment of stereo recorders requires multiple trips to the wetland. These costs could be avoided by increasing the battery life (i.e., adding an external power source) and data storage capacity of stereo recorders, as well as adding timers. For the sample regime tested above this would amount to a 4% decrease in costs ($1,150). In addition, there are three considerations to be made if the STEREO‐AUDIBLE option tested in this study is used to index number of individuals.

First, the strength of the relationship between numbers of calling bitterns detected using STEREO‐AUDIBLE options and those detected using field observers (OBS) could vary when different processors are being used. In this study, we found that the process of deciding direction and volume used to distinguish calls from individual bitterns was subjective, requiring expertise and high concentration. Results here were based on one sound file processor but feedback from coworkers that also tried the method suggested that variability in results may be high across sound file processors. The extent of this variability would need to be determined before this method could be pursued further.

Second, the ability to distinguish bitterns using the criteria outlined in this study may vary with the number of bitterns calling. For example, the criteria (three volume classes and two direction classes) only provides six potential outcomes, limiting the maximum number of bitterns distinguishable to six. This restriction is unlikely to have affected this study because more than six bitterns were only detected by field observers during one count. However, had the study been conducted in 2009, when as many as twelve bitterns were heard calling within similar count times (Williams, 2016), results may have been different.

Third, the ability to distinguish birds will be affected by the bird's location in relation to the recording device. For example, to be able differentiate between as many as six bitterns, birds would need to be distributed evenly across the 180° trajectory in front of the recorder (e.g., Figure 2). This situation is unlikely to arise at all monitoring sites, as wetlands vary in their topography. In larger wetlands, recorders are more likely to be deployed with birds spread around the full 360° radius (e.g., in the middle of a wetland), and many smaller wetlands do not have uniformly linear edges meaning recorders are equally likely to be deployed on corners of the wetland (<90°).

Many of these challenges could be remedied if stereo options are used in conjunction with techniques that can provide additional information to narrow estimates of number of individuals (i.e., vocal individuality; Gilbert et al., 1994). However, to achieve this with the stereo options trialed in this study it is important to be aware that Olympus LS‐30 recorders record in a compressed format (mp3), which contains less information about call characteristics compared with WAV‐storing counterparts (such as our MONO options) (Brandes, 2008; Obrist et al., 2010). If this had had any effect on call detection in this study, we would have observed a lower comparability between STEREO options and observers compared with the comparability of MONO with observers. As it happens there was little difference in this regard. This may be because mp3 compression has been designed to minimize loss of data in the range of human hearing (Brandes, 2008; Rempel, Hobson, Holborn, Van Wilgenburg, & Elliott, 2005), suggesting it is suitable for use when the objectives are to replace people with the devices, as in the case of this study. However, in cases where intricate sound file analysis is required (i.e., to measure vocal individuality) options that use compressed formats will be less desirable (Brandes, 2008; Obrist et al., 2010).

If call‐rate is sufficient as an index of relative abundance alone (i.e., no additional information or public involvement is desired) then the 73% or 50% savings incurred using the mono‐visual option over observers and stereo‐audible options would be preferred (MONO‐VISUAL = $NZ 13,925; STEREO‐AUDIBLE = $28,113; OBS = $51,643). Like all index measures, calls per unit time is only useful as a measure if it correlates to abundance (Caughley, 1977). Results from other studies are mixed with this regard, with some reporting that call‐rate measured using recorders correlates strongly with abundance (Payne, Thompson, & Kramer, 2003), and others reporting that recorders are uninformative in relation to abundance (Cunningham, Lindenmayer, & Lindenmayer, 2004). In the case of Australasian bitterns, it is likely that call‐rate derived from recorders will be informative in terms of male bittern abundance because calling‐rate is known to be predictable, and the relationship between the number of calls heard and the number of calling individuals detected is strong (Williams, 2016).

In addition, the use of recording devices creates opportunities to obtain density from other techniques (e.g., combinations of call characteristics and spatial information, and array‐based sampling; Brandes, 2008; Dawson & Efford, 2009; Efford, Borchers, & Byrom, 2009; Mennill, Battiston, Wilson, Foote, & Doucet, 2012; Marques et al., 2013; Stevenson et al., 2015). However, for these techniques to be useful for bitterns, more information is required about the effective sampling area of recorders. In addition, for recorders to be useful on a national scale, a method of processing large volumes of sound files in a cost‐effective, timely manner would be required. Several studies have shown that automating the process of detecting calls using software packages has potential in this regard (Bardeli et al., 2010; Brandes, 2008; Digby, 2013; Graff, 2014; Steer, 2010). Nevertheless, more comparative work is required before the cost‐effectiveness of these tools can be determined. Joshi et al. (2017) showed that such software is cost‐effective in some situations but not others. Automatic detection of bittern calls has so far not been found to be cost‐effective, either because expensive recording equipment was needed (Frommolt & Tauchert, 2014) or because it gave high false positive rates (21% precision rate; Priyadarshani, 2017). However, work on this is ongoing.

## 5 CONCLUSIONS

In the case presented here, Australasian bitterns were a challenge to monitor due to several cryptic‐species‐specific characteristics and a few site‐specific logistic constraints. The first of these challenges was solved by showing that calling‐rate is predictable in terms of time of day, time of year, and various weather conditions (Williams, 2016). However, this research also shows that bitterns should ideally be monitored during a short sampling window starting one and a half hours before sunrise and ending thirty min before sunrise, in September and October. Monitoring using observers at this time at some sites is not feasible. For example, people cannot count all locations at sites that are large and difficult to access within tight windows and there are health and safety implications associated with having staff or volunteers out at these times. In this study, we demonstrate that recording devices not only solve our spatial site‐specific constraint, but using timers and recording devices, multiple locations can now be sampled concurrently at “optimum” times, therefore solving any additional temporal restrictions. Costs presented here were in New Zealand Dollars and depend upon multiple factors, such as: product availability and currency exchange rates at time of purchase. As a result, costs may differ across studies and between countries. However, the approximate differences and ranking of different methods presented here should apply to any projects that target a species with similar cryptic characteristics and site‐specific logistic constraints.

## CONFLICT OF INTEREST

None declared.

## AUTHOR CONTRIBUTION

EMW and COD conceived and designed the field component comparing recorder options, while EMW and DPA conceived and designed the cost‐benefit analysis. EMW conducted the fieldwork with input from COD and DPA and assistance from acknowledged field staff. EMW analyzed the sound files with advice from others working in similar fields (acknowledged). All authors contributed to the writing of the manuscript and gave final approval for publication.

## DATA ACCESSIBILITY

The data used in this manuscript are available at request from Department of Conservation archives.
